# Structure of BTK kinase domain with the second-generation inhibitors acalabrutinib and tirabrutinib

**DOI:** 10.1371/journal.pone.0290872

**Published:** 2023-08-31

**Authors:** David Y. Lin, Amy H. Andreotti

**Affiliations:** Roy J. Carver Department of Biochemistry, Biophysics and Molecular Biology, Iowa State, University, Ames, IA, United States of America; Concordia University, CANADA

## Abstract

Bruton’s tyrosine kinase (BTK) is the target of the therapeutic agent, Ibrutinib, that treats chronic lymphocyte leukemia (CLL), mantle cell lymphoma (MCL) and other B cell malignancies. Ibrutinib is a first in class, covalent BTK inhibitor that limits B-cell survival and proliferation. Designing new inhibitors of BTK has been an important objective for advancing development of improved therapeutic agents against cancer and autoimmune disorders. Based on the success of Ibrutinib, several second-generation irreversible BTK inhibitors have been developed that exhibit fewer off-target effects. However, the binding-mode and their interaction with Btk have not been experimentally determined and evaluated at atomic resolution. Here we determined the first crystal structure of the BTK kinase domain in complex with acalabrutinib. In addition, we report a structure of the BTK/tirabrutinib complex and compare these structures with previously solved structures. The structures provide insight in the superior selectivity reported for acalabrutinb and guide future BTK inhibitor development.

## Introduction

Bruton’s tyrosine kinase (BTK), is a non-receptor tyrosine kinase that is one of the five TEC family kinases expressed primarily in hematopoietic cells. In addition to BTK, the TEC family includes ITK, TEC, BMX, and TXK [1], which all function in signaling pathways downstream of immune cell receptors. BTK propagates signaling from the B-cell receptor, including B cells at very early stages of differentiation, as well as the Fc receptor, Toll-like receptors and chemokine receptors. The discovery of the immunodeficiency disease, X-linked agammaglobulinemia (XLA), and subsequent findings that showed genetic defects in the *Btk* gene cause XLA [2], cemented the importance of this kinase in B cell signaling cascades. In addition to its causative role in XLA, BTK is an essential signaling molecule for proliferation of leukemia and lymphoma cells in B cell malignancies. As a result, BTK is a target for clinical intervention for diseases such as mantle cell lymphoma (MCL), chronic lymphocytic leukemia (CLL), Waldenström macroglobulinemia (WM), small lymphocytic lymphoma, marginal zone lymphoma, and chronic graft-versus-host disease [3–10]. Clinical application of BTK inhibitors in inflammatory and autoimmune disease is also being actively explored [11, 12].

The first BTK inhibitor approved for use in the clinic was Ibrutinib; a small molecule initially described as a high affinity BTK inhibitor [13] and subsequently shown to be effective in animal models of autoimmune disease and B-cell lymphoma [14]. Ibrutinib forms a covalent bond with the cysteine 481 (C481) side chain in the BTK active site resulting in irreversible, sustained inhibition of kinase activity. In 2013, 2014 and 2015 the US Food and Drug Administration approved ibrutinib for the treatment of MCL, CLL, and WM, respectively. More recently, the FDA approved the combination of Ibrutinib with rituximab (a monoclonal antibody targeted against the B cell surface antigen CD20) for CLL [15, 16]. In the decade since Ibrutinib received FDA approval, it and other BTK inhibitors are now broadly used in cancer treatment providing patients with excellent strategies to manage B cell malignancies.

**Fig 1:**
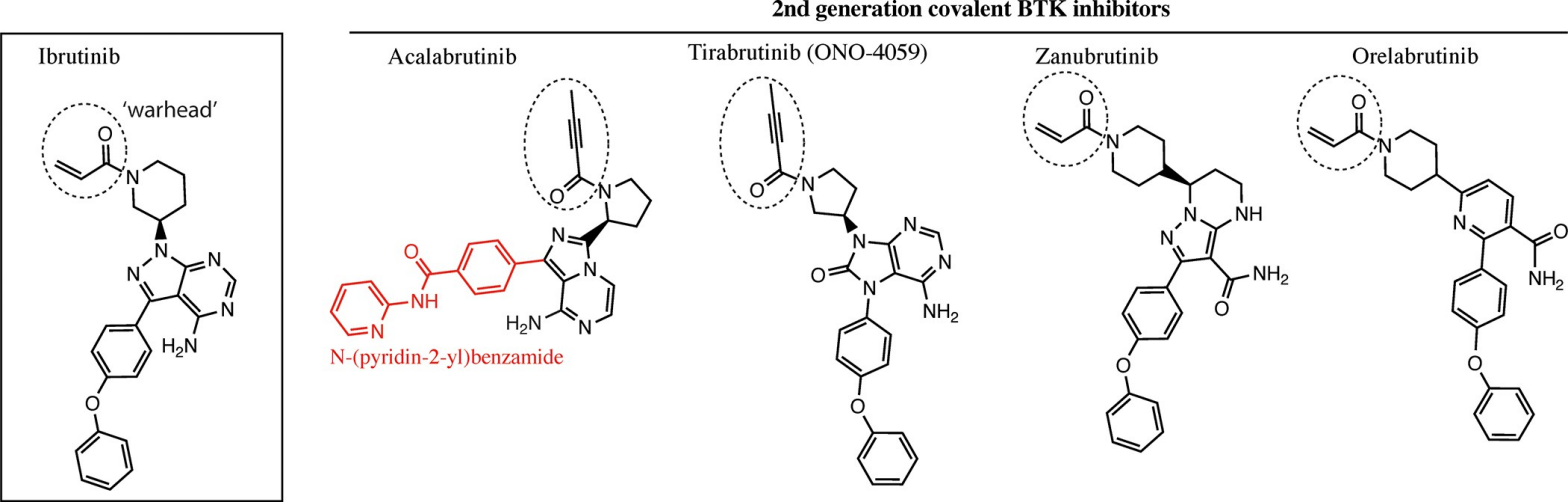
The chemical structures of irreversible BTK inhibitors.

Off target side effects and acquired resistance to Ibrutinib [17–22] has spurred the development of second-generation, covalent BTK inhibitors [23, 24]. Acalabrutinib, Zanubrutinib and Orelabrutinib [25] are approved for clinical use in the United States. Another second-generation inhibitor, Tirabrutinib, is not yet FDA approved but is in clinical use in Japan and Taiwan [26]. Ibrutinib and its related second-generation inhibitors (**Fig 1**) are irreversible covalent drugs and of those approved by the FDA, Ibrutinib is the most potent, but acalabrutinib and zanubrutinib are characterized by higher specificity (lower off-target rates) and fewer adverse side effects [24, 27]. Several outstanding reviews describing development in the BTK inhibitor arena are available [3, 28–30].

High-resolution structures of the BTK kinase domain bound to covalent, active site inhibitors provide valuable insight into mechanisms of action and specificity. Indeed, the development of Zanubrutinib [31, 32] is an excellent example of the value of kinase/drug co-crystal structures combined with SAR (structure activity relationship) analyses to yield compounds with improved specificity. High-resolution structures of BTK kinase domain/drug co-complexes are currently available for Ibrutinib (PDB: 5p9j), Zanubrutinib (PDB: 6j6m), and Tirabrutinib (PDB: 5p9m) but not for BTK bound to Acalabrutinib or Orelabrutinib. Of the second generation BTK inhibitors, Zanubrutinib and Orelabrutinib share the acrylamide warhead of Ibrutinib while Acalabrutinib and Tirabrutinib contain a butynamide warhead (**Fig 1**). Here we reported the first structure of acalabrutinb in complex BTK as well as a new tirabrutinib:BTK complex structure that differs from the previously published structure of BTK bound to tirabrutinib (PDB: 5p9m).

## Materials and methods

### Cloning, expression, and purification

The kinase domain (KD) of murine BTK (residues 396–659) was cloned into pET28b vector with a C-terminal 6xHistidine tag. Several mutations were made to the construct including a K430R mutation to stabilize the inactive conformation, six mutations in the activation loop of BTK with the corresponding ones in Itk (L542M S543T, V555T, R562K, S564A and P565S) and a Y617P mutation for bacterial expression (BTK KD). For protein expression, the plasmid was transformed to BL21 (DE3) cells (Millipore Sigma). Cells were grown in LB broth (Fisher Scientific) supplemented with 50 μg/mL kanamycin (Sigma) at 37°C until the optical density at 600 nm reached ~0.6. Protein expression was induced with 1 mM isopropyl β-d-1-thiogalactopyranoside (IPTG) for 24 hours at 18°C. Cells were harvested by centrifugation at 3,000 × g for 20 min, resuspended in lysis buffer (50 mM Tris pH 8.0, 500 mM NaCl and 10% glycerol), and stored at -80°C until use. Cells were thawed with the addition of 0.5mg/ml lysozyme and 1500U of DNAse I (Sigma) and lysed by sonication on ice at 50% amplitude for 2 min with 0.5-second on and 1.5-second off intervals. The lysate was clarified by centrifugation at 20,000 × g for 1 hour at 4°C, and the supernatant was applied to a Ni-NTA column (QIAGEN) equilibrated with lysis buffer. The column was washed with 20 column volume of lysis buffer with 20mM imidazole and eluted with lysis buffer with 300 mM imidazole. The sample was concentrated, and further purified with HiLoad 26/600 Superdex 75 pg (Cytiva) with FLPC buffer (20 mM Tris pH 8.0, 150 mM NaCl and 10% glycerol). Purified proteins were concentrated to 20 mg/mL, flash-frozen in 100ul/aliquot with liquid nitrogen, and stored at -80°C.

### Crystallization

Concentrated BTK KD proteins were thawed and mixed with a final concentration of 1mM acalabrutinib or tirabrutinib (ONO-4059) (Selleckchem) and 10% DMSO for 30min on ice. Crystallization screening were carried out with mosquito® (SPT Labtech) by mixing 0.1uL of BTK KD proteins with 0.1 uL of pre-chilled crystallization solution in sitting-up INTELLI plates (Art Robbins Instruments). The crystallization plates were then transferred and stored at 4°C. Btk KD/acalabrutinib crystals were obtained in the condition of 20% PEG 3350, 0.1M Bis-tris propane, pH 6.5, and 0.2M sodium bromide. Btk KD/tirabrutinib crystals were obtained in the condition of 16% PEG 8000 and 0.1M sodium citrate, pH 5.5. The crystals were harvested with a cryo-protectant solution (crystal conditions + 20% glycerol) and flash-frozen in liquid nitrogen.

### Data collection, structure determination, and refinement

X-ray diffraction data were collected at the Advanced Photon Source (APS) beamline 24-ID-E NE-CAT (Center for Advanced Macromolecular Crystallography). Data sets were indexed, merged, and scaled using autoPROC (Global Phasing) [33–35]. The structures were solved by molecular replacement using Phaser in the CCP4i suite [36, 37] using one protein monomer from the ibrutinib-bound KD structure (PDB code 5P9J) as the search model. JLigand in the CCP4i suite and ELBOW in Phenix suite was used to generate the restraints for acalabrutinib and tirabrutinib [38, 39]. The structures were refined using Phenix [40] and built in Coot [41]. Complete X-ray collection and refinement statistics are provided in **Table 1**. The figures are prepared with Pymol [42]. PDB codes for the structures solved here are as follows: BTK KD/acalabrutinib: 8FD9, BTK KD/tirabrutinib: 8FF0.

**Table 1:** Data collection and refinement statistics (molecular replacement).

	Btk/acalabrutinib		Btk/tirabruitinib
**Data collection**			
Space group	P 21 21 21		P61
Cell dimensions			
*a*, *b*, *c* (Å)	42.92, 50.31, 123.48		108.18, 108.18, 45.17
α, β, γ (°)	90, 90, 90		90, 90, 120
Resolution (Å)	61.75–1.626 (1.745–1.626) *		19.409–2.60 (2.71–2.60) *
*R*_merge_	0.148 (1.276)		0.064 (2.049)
*I* / σ*I*	8.9 (1.5)		17.2 (1.1)
Completeness (%)	82.6 (21.8)		93.5 (44.0)
Redundancy	5.5(5.4)		9.9 (9.9)
**Refinement**			
Resolution (Å)	19.74–1.70		19.41–2.60
No. reflections	27871 (1462)		8798 (362)
*R*_work_ / *R*_free_	0.1866 / 0.2276		0.2410 / 0.2667
No. atoms	4758		3957
Protein	4428		4270
Ligand/ion	63		1
Water	267		6
*B*-factors			
Protein	24.778		110.65
Ligand/ion	19.642		95.849
Water	30.549		81.623
R.m.s. deviations			
Bond lengths (Å)	0.002		0.003
Bond angles (°)	0.474		0.805

*Values in parentheses are for highest-resolution shell.

## Results

### Construct design and crystallization of inactive BTK kinase domain

To facilitate crystal packing, we expressed the kinase domain of murine BTK (residues 396–659) with a kinase-inactive mutation, K430R, and substituted six residues in the activation loop with the corresponding residues from ITK (hereafter referred to as BTK KD), which we have previously shown stabilizes the activation loop in solution [43] and has been employed in prior crystallization efforts [44]. Cocrystal screening was pursued with purified inactive BTK KD in the presence of acalabrutinib (or tirabrutinib). BTK KD/acalabrutinib crystals appeared in multiple conditions with 20% PEG 3350, 0.1M Bis-tris Propane, pH 6.5–7.5 and 0.2M sodium fluoride, sodium bromide, or sodium iodide. The x-ray data was collected from the crystals that appeared in the condition with 20% PEG 3350, 0.1M Bis-tris propane, pH 6.5 and 0.2M sodium bromide.

### Complex crystal structure

**Fig 2:**
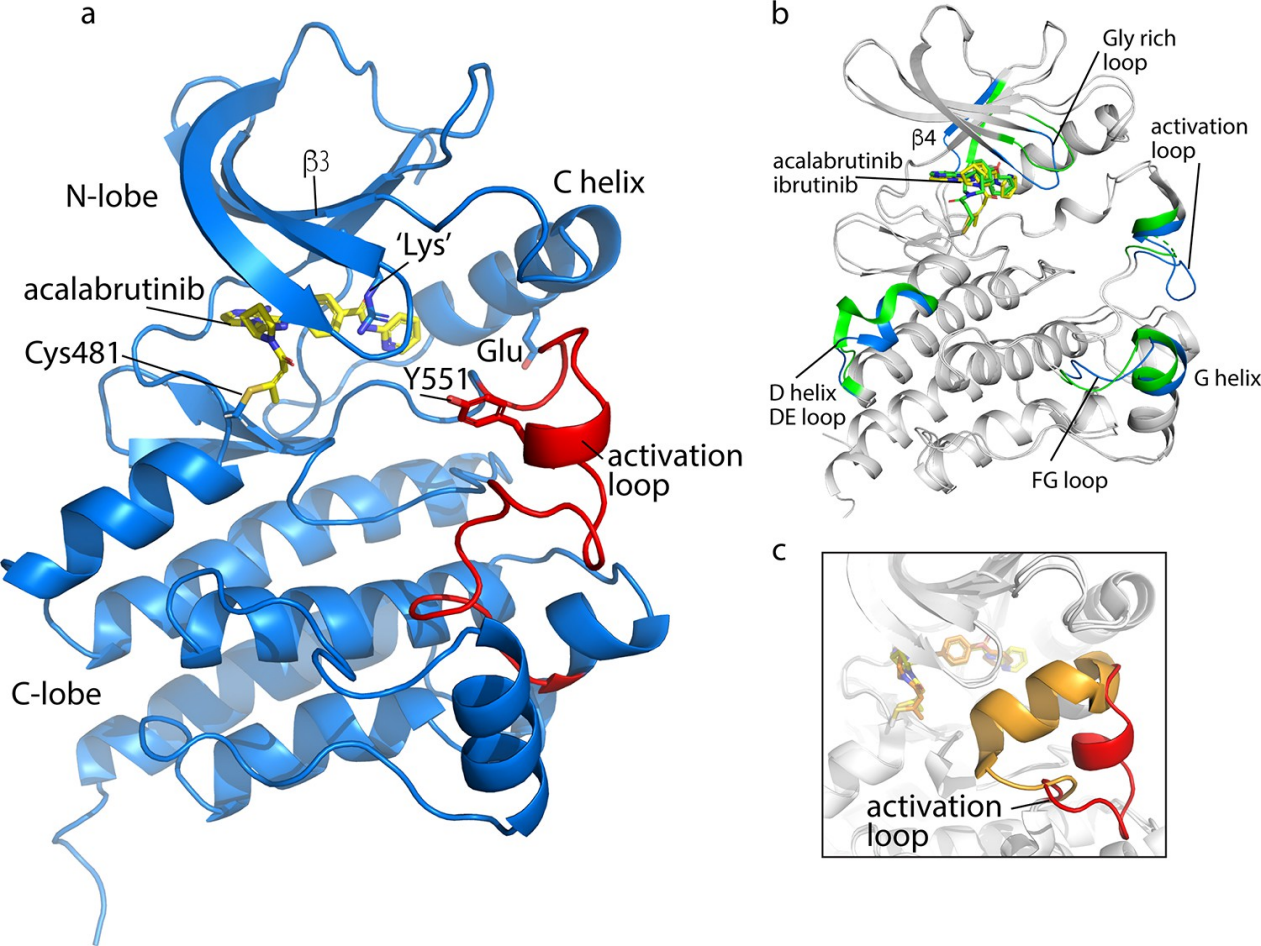
The acalabrutinib-bound BTK kinase domain. a. Overall structure of the BTK KD/acalabrutinib complex. Key features of the kinase domain are labeled. Acalabrutinib is shown in yellow sticks and covalently attached to Cys481. Activation loop is colored red with Tyr551 shown in sticks. The structure represents an inactive kinase as the C-helix adopts the ‘out’ position moving the conversed Glu away from the conserved Lys on β3 shown in sticks. Since the structure was solved with the Lys430Arg mutation, the conserved lysine is indicated as ‘Lys’. b. Superpositions of the acalabrutinib- and the ibrutinib-bound Btk kinase domains. Acalabrutinib and ibrutinib are shown in yellow and green sticks respectively. The regions which display differences between the two structures are colored in blue (acalabrutinib) and green (ibrutinib). c. Conformations of the activation loops in the BTK KD/acalabrutinib complex (red) and the previously solved tirabrutinib-bound structure (orange, PDB: 5P9M).

The structure of the BTK KD/acalabrutinib complex was determined to 1.7Å (**Fig 2A**, **Table 1**). The structure of the BTK/acalabrutinib complex exhibits the expected kinase domain bilobed structure connected with a hinge region (**Fig 2A**). The ATP binding cleft is formed between the two lobes, and the active site cysteine (Cys481) is in the hinge region on the edge of the ATP binding pocket. The kinase domain exhibits the “C-helix out” inactive conformation resulting in separation of the conserved Lys/Glu pair as observed in many previous BTK KD structures bound to inhibitors. The activation loop is ordered and adopts the so-called “DFG-in” conformation and the activation loop tyrosine (Tyr551) points toward the active site.

### Comparison of available BTK/inhibitor complexes

Comparing the BTK KD/acalabrutinib complex to the previously solved BTK KD/ibrutinib complex structure (PDB: 5P9J) reveals a root-mean-square deviation (RMSD) of 0.7933 Å (**Fig 2B**). The BTK KD/zanubrutinib structure (PDB: 6J6M) is nearly identical to BTK KD/ibrutinib and so only comparison of the new BTK KD/acalabrutinib complex to BTK KD/ibrutinib is shown. The six mutated activation loop residues along with the K430R mutation did not display any major conformational change with a combined RMSD of 0.3360 Å. The regions with the largest RMSD differences (colored in **Fig 2B**) include the glycine-rich loop (residues 410 to 414), the β4 strand (residues 460–463), the end of the D-helix and DE loop (residues 487 to 494), the activation loop (residues 550 to 558), and the FG loop into the G helix (residues 598 to 607).

The other BTK inhibitor for which a complex structure is available is tirabrutinib (PDB: 5P9M) [45]. Comparison of the BTK KD/acalabrutinib complex structure with the tirabrutinib bound BK KD shows large differences in the conformation of the activation loop (**Fig 2C**). This is accompanied by extensive contacts between the side chain of Phe413 on the glycine rich loop and the activation loop that are not present in the acalabrutinib complex structure or any of the other inhibitor bound structures. To test the degree to which this unusual activation loop conformation (**Fig 2C**) might be related to tirabrutinib binding, we solved the structure of BTK KD/tirabrutinib complex structure via co-crystallization of BTK and tirabrutinib (**S1 Fig**). Co-crystallization (as opposed to the “soaked” BTK/tirabrutinib complex solved previously [45]) yielded crystals of the BTK KD/tirabrutinib complex in 18% PEG3350 or PEG 8000, 0.1M sodium citrate tribasic dihydrate, pH5.5. The x-ray data was collected from the crystals in the PEG 8000 condition and the BTK KD/tirabrutinib complex structure was determined to 2.6Å. Tirabrutinib binding is identical in the two structures but the activation loop adopts very different conformations in the two structures (**S1A Fig**). The activation loop conformation in our co-crystallized BTK KD/tirabrutinib complex is nearly identical to structures of the BTK kinase domain bound to ibrutinib, zanubrutinib and acalabrutinib (**S1B Fig**). The RMSD for the activation loop residues 539–567 in our BTK KD/tirabrutinib structure the BTK KD complex with ibrutinib is 0.43 Å, with zanubrutinib is 0.58 Å, and with acalabrutinib is 0.46 Å. Thus, we interpret differences in the tirabrutinib complex we report here and that solved previously to suggest that the drug bound kinase domain undergoes dynamic fluctuations. Indeed, the activation loop conformation in the previous BTK KD/tirabrutinib structure is characterized by elevated B-factors compared to the rest of the structure and some side chain densities are not visible. It will be important to study these complexes in solution to determine the extent to which kinase dynamics differs for the different inhibitors.

### Inhibitor binding

**Fig 3:**
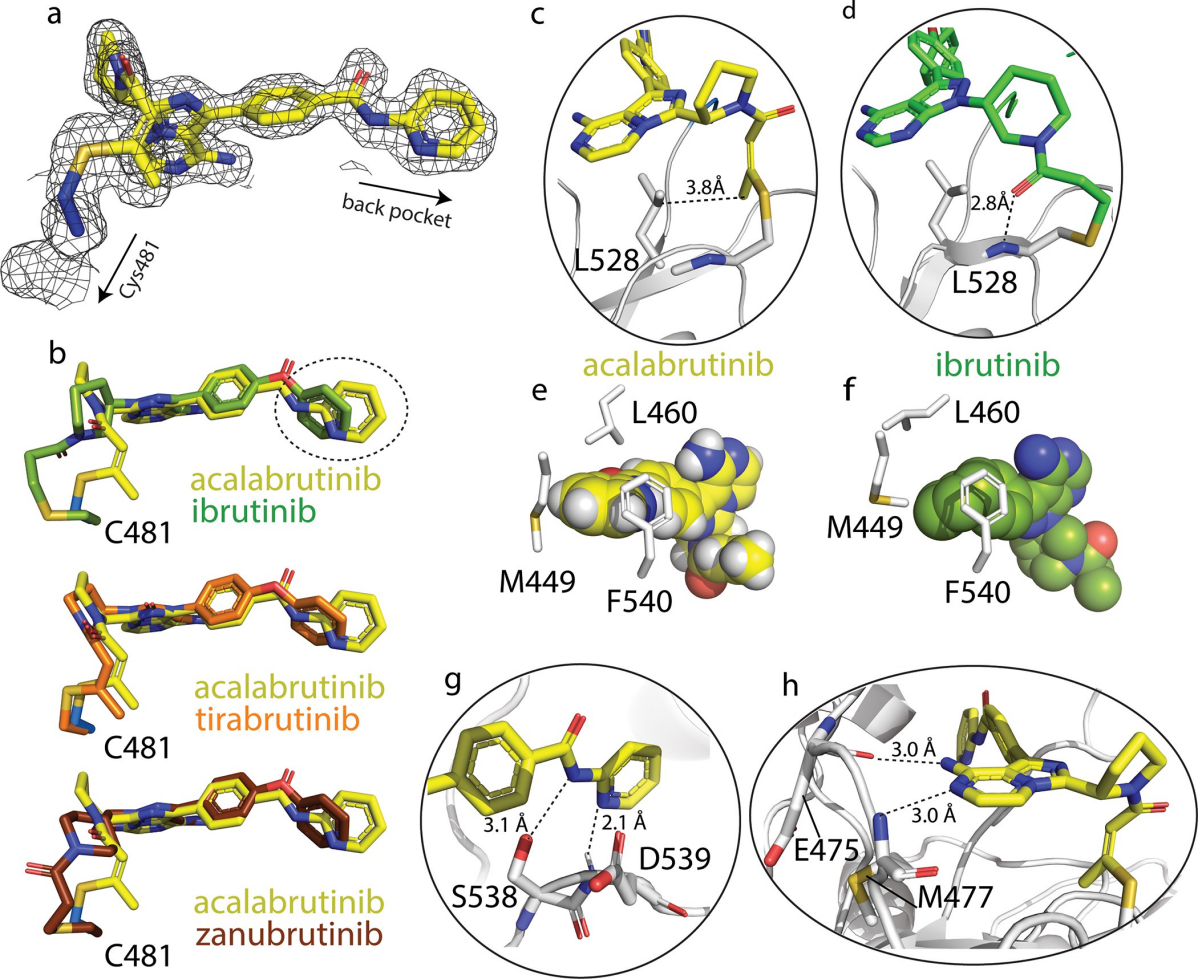
Binding modes of covalent BTK inhibitors. a. 2Fo-Fc density map of acalabrutinib at the binding site. The locations of the back pocket and Cys481 are indicated. b. Active site bound acalabrutinib (yellow), superimposed with ibrutinib (green), tirabrutinib (orange) and zanubrutinib (brown). c. and d. Close-up views of the covalent attachment of acalabrutinib (c) and ibrutinib (d) to Cys481 in the BTK active site. The interaction of each drug with Leu528 is shown. e. and f. Close-up view of acalabrutinib and ibrutinib binding to the back pocket. Acalabrutinib and ibrutinib are shown in space-filling model and side chains of Met449, Leu460, and Phe540 are shown in white sticks. g. and h. Hydrogen bonding interactions between acalabrutinib and Ser538, Asp539, Glu475, and Met447.

The electron density map clearly reveals the position of acalabrutinib and the covalent bond formed between inhibitor and the Cys481 thiol group (**Fig 3A**). Specific contacts between acalabrutinib and BTK are listed in **Table 2**. The overall shape of the acalabrutinib molecule bound to the BTK active site is similar to that of bound ibrutinib, tirabrutinib, and zanubrutinib despite differences in the chemical structures (**Figs 1 and 3B**). Differences are noted at the site of covalent attachment to the Cys481 side chain. Acalabrutinib and tirabrutinib both contain the butynamide ‘warhead’ instead of the acrylamide group found in ibrutinib, zanubrutinib and orelabrutinib. Comparing the BTK/acalabrutinib complex structure to the other available structures reveals that Cys481 adopts distinct sidechain rotamers; acalabrutinib and tirabrutinib overlay closely while the ibrutinib and zanubrutinib structures show that the Cys481 side chain is rotated ~120° (**Fig 3B**). These differences at the site of covalent attachment to each of the active site inhibitors suggest a degree of flexibility in this region of BTK to accommodate drug binding. Moreover, the differences between the warheads of acalabrutinib versus ibrutinib and the different trajectory each drug takes from the covalent attachment site result in distinct contacts between drug and kinase domain residues. In the acalabrutinib bound structure, the side chain of Leu528 is in close contact with the methyl group in the Michael adduct (**Fig 3C**) whereas the ibrutinib structure shows a hydrogen bond between the carbonyl oxygen of the drug and the backbone amide of Cys481 (**Fig 3D**).

**Table 2:** List of contacts between Btk residues and acalabrutinib atoms that are within 4 Å, and the interaction types: Main chain (mc) or side chain (sc) interactions.

Btk	Interaction type	Acalabrutinib
L408	mc, sc	C2, C3, C9, C12
G409	mc	C3
T410	mc	C4
G411	mc	C4, C7, O1
V416	mc, sc	C3, C4, C5, C6, C8, C11, C15, N2, N7
A428	mc, sc	C6, C10,C15, N3, N4
R430	mc, sc	C14, C16, C17, C18, C20, C22, O2, N5, N6
M449	mc, sc	C24, C25, C26
458	sc	N4
L460	sc	C22, C23, C24, C25, C26, N5, N6
I472	mc, sc	C20, C25, O2, N6
T474	mc, sc	C11, C15, C17, C18, C20, O2, N3, N4, N5
E475	mc	C10, N4
Y476	mc, sc	C12,N3
M477	mc, sc	C10, C12, N3, N4
G480	mc	C9, C12
C481	mc, sc	C1, C7, C13, C19, C20, C21, N1
R525	mc	C21
C527	mc	C21
L528	mc, sc	C1, C5, C6, C8, C9, C10, C11, C12, C13, C14, C16, C19, C21, N3, N4, N7
S538	mc, sc	C18, C20, C22, C23
D539	mc, sc	C20, C22, C23, C24, N5, N6
F540	mc, sc	C24
M542	mc, sc	C25, C26, N6

The opposite end of acalabrutinib contains the slightly longer N-(pyridin-2-yl) benzamide in place of the 4-phenoxyphenyl group in the other inhibitors causing an extension into the ‘back pocket’ [46] of the BTK kinase domain. The deeper binding pose of acalabrutinib is accompanied by close contacts between acalabrutinib and the side chains of Leu460 and Met449 (**Fig 3E).** More specifically, Leu460 in the BTK/acalabrutinib complex points its side chain directly into the center the pyridine group forming strong CH-π interactions, which might indicate a more important role of Leu 460 in binding to acalabrutinib. In contrast, ibrutinib (and the other 4-phenoxyphenyl containing inhibitors (**Fig 1**)), do not create as favorable Van der Waals contacts with the L460 and M449 side chains (**Fig 3F**). Finally, we note that the structure of acalabrutinib bound to the BTK active site corresponds well with the model generated by Barf et al [47]. Drug orientation is the same and hydrogen bonds proposed in the model are observed in the crystal structure (Fig 3G, 3H).

## Discussion

The structures of the BTK KD/acalabrutinib and BTK KD/tirabrutinib complexes presented here add to existing structures of BTK bound to the different covalent inhibitors in use in the clinic to treat B cell malignancies. These structures are important as efforts continue to develop potent BTK inhibitors that are characterized by fewer adverse effects. Because the N-(pyridin-2-yl)benzamide group in acalabrutinib is unique among the covalent inhibitors (**Fig 1**), the ability of acalabrutinib to have less off-target effects could arise from distinct interactions within the BTK active site, especially contacts between the pyridine group and specific side chains of BTK (**Fig 3E**). To explore this idea, we aligned sequences of ibrutinib off-target kinases, the other TEC kinases, Src, Csk, EGFR, Erbb and Jak3 and examined sequence conservation of the BTK residues involved in acalabrutinib binding (**S2A and S2B Fig**). The five residues forming the narrow part of the back pocket in BTK, Met542 (Leu in human BTK), Leu460, Met449, Phe540 and Ile472, are identical or conserved (**S2A and S2B Fig**). The exceptions are BMX and JAK3, where a phenylalanine in BMX and a tyrosine in Jak3 are in place of the Leu460. BMX displayed a nearly 10 fold decrease of inhibition by acalabrutinib (BMX IC50 of ~46nM versus BTK at 5.1nM ((1)). Jak3, in contrast, is not inhibited by acalabrutinb (IC50 of >1000 ((1)). In structures of the JAK3 kinase domain, Tyr886 (corresponds to BTK Leu460) points away from the back pocket of the active site suggesting that acalabrutinib binding to JAK3 might be destabilized due to loss of favorable contacts. However, the otherwise high sequence conservation within the active site of off-target kinases (**S2A Fig**), and the observation that acalabrutinib does not inhibit EGFR or the closely related ITK [47], suggests the improved selectivity of acalabrutinib is probably not determined by the unique N-(pyridin-2-yl)benzamide group binding to the back pocket.

The BTK/acalabrutinib crystal structure reported here closely matches a model of the bound inhibitor generated by Barf et al. [47]; the predicted hydrogen bonds between acalabrutinib and BTK are observed in our crystal structure. In that study, the authors also provide an explanation for the observation that acalabrutinib exhibits higher selectivity to some cysteine-containing kinases over others. They suggest that the combined effects of the lower electrophilicity of the butynamide ‘warhead’ (compared to the acrylamide functional group) with a lower pKa for the nucleophilic thiol group explain acalabrutinib’s selectivity. Specifically, the presence in BTK of an asparagine in the i+3 position relative to Cys481 results in a lower pKa for the Cys481 thiol group and hence higher nucleophilicity that compensates for the lower electrophilicity of the butynamide ‘warhead’. As a result, BTK (but not kinases such as ITK, EGFR and JAK3 that contain an aspartate in the i+3 position (**S2A Fig**)) effectively captures the butynamide containing inhibitor while the kinases that contain a less nucleophilic cysteine side chain do not. The crystal structure of acalabrutinib bound to BTK reported here lends further experimental support to the model of Barf et al.

The importance of the local environment surrounding Cys481 and the chemical nature of the ‘warhead’ may contribute to drug specificity. However, we note that the related TEC family kinases, TEC, BMX and TXK, share the asparagine sidechain of BTK in the i+3 position (**S2A Fig**) and yet acalabrutinib does not inhibit these kinases as effectively [48–50]. There may therefore be additional structural or dynamic features present in these full-length kinases that influence inhibitor specificity and/or rate of covalent capture. Sequence alignment of the TEC family kinases (only ITK contains the aspartate at i+3) shows several regions with little to no sequence similarity across the family (red boxes in **S2A Fig**). Notably, the primary sequence following C481 exhibits one of the longest continuous stretches of sequence disparity across the five kinases. Interestingly, this region and the others with high sequence dissimilarity cluster in loops on the distal side of the kinase domain (opposite of the activation loop), which may indicate distinct dynamics through the hinge region and into the active site (**S2B Fig**). In addition, the regions of largest sequence differences among the TEC family kinases surround the catalytic spine (C spine), which includes bound drug (**S2B Fig**). Further work and a focus on protein dynamics will be necessary to fully dissect and eventually exploit the various structural, dynamic, and chemical contributions to drug specificity.

## Conclusion

This study reports the structures of murine BTK kinase domain with acalabrutinib and tirabrutinib bound to the active site. Acalabrutinib binds deeply into the hydrophobic back pocket of the BTK active site with its unique N-(pyridin-2-yl)benzamide group making extensive contacts with BTK side chains. While this inhibitor is distinct from ibrutinib and other second-generation inhibitors, it is likely that the unique N-(pyridin-2-yl)benzamide is not the primary source of the improved selectivity of acalabrutinib. Indeed, this binding pocket is highly conserved pocket among off-target kinases making specific inhibitor design quite challenging. However, the localized nature of the sequence differences among the TEC family point to the possibility that differential kinase dynamics may extend into the active site to influence drug binding specificity. As well, the previously described arguments of balanced electrophilicity/nucleophilicity of the warhead and cysteine thiol, respectively as a driving force for target selectivity are very appealing [47].

## Supporting information

S1 Fig Comparison of BTK/acalabrutinib and BTK/tirabrutinib complexes. a. Superposition of the BTK KD/tirabrutinib structure solved here with the previously solved structure (PDB code: 5P9M) showing the different activation loop conformations as in Fig 2c. b. Superposition of the BTK KD/acalabrutinib (blue) and BTK KD/tirabrutinib (white) structures solved here.
(TIF)

S2 Fig Sequence Alignment of Ibrutinib off-target kinases. a. Sequence alignment of eleven protein kinases; the five TEC family kinases are listed above the horizontal line. Cys481 (BTK numbering) and the i+3 position are indicated with black and blue boxes, respectively. Residues that surround the active site and directly contact bound drug are in red with yellow highlight. Red boxes indicate regions of low sequence similarity among the TEC kinases. Asterisks indicate C spine residues. b. Structure of the BTK KD/acalabrutinib complex showing the cluster of residues with low sequence conservation in the TEC family (red spheres). Black spheres show the conserved residues of the C spine and bound acalabrutinib is shown in yellow spheres.
(TIF)

S1 File (PDF)

S2 File (PDF)
