# Supplementary figures and images for: Structure of BTK kinase domain with the second-generation inhibitors acalabrutinib and tirabrutinib
Source: PLoS One. 2023 Aug 31;18(8):e0290872. doi: 10.1371/journal.pone.0290872 (PMC10470882; doi:10.1371/journal.pone.0290872)

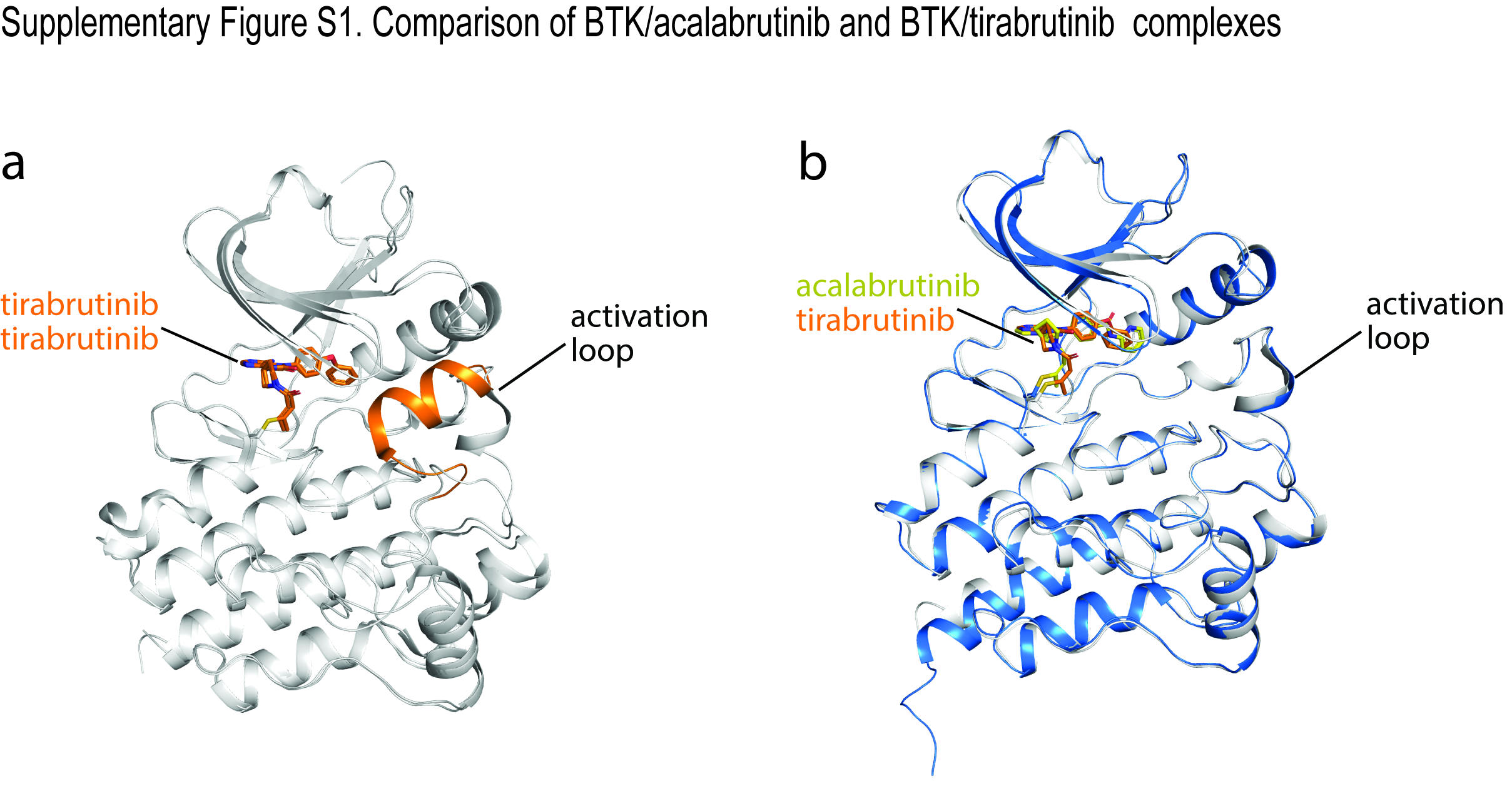

Supplement: S1 Fig — a. Superposition of the BTK KD/tirabrutinib structure solved here with the previously solved structure (PDB code: 5P9M) showing the different activation loop conformations as in Fig 2c. b. Superposition of the BTK KD/acalabrutinib (blue) and BTK KD/tirabrutinib (white) structures solved here. (TIF) [file pone.0290872.s001.tif]

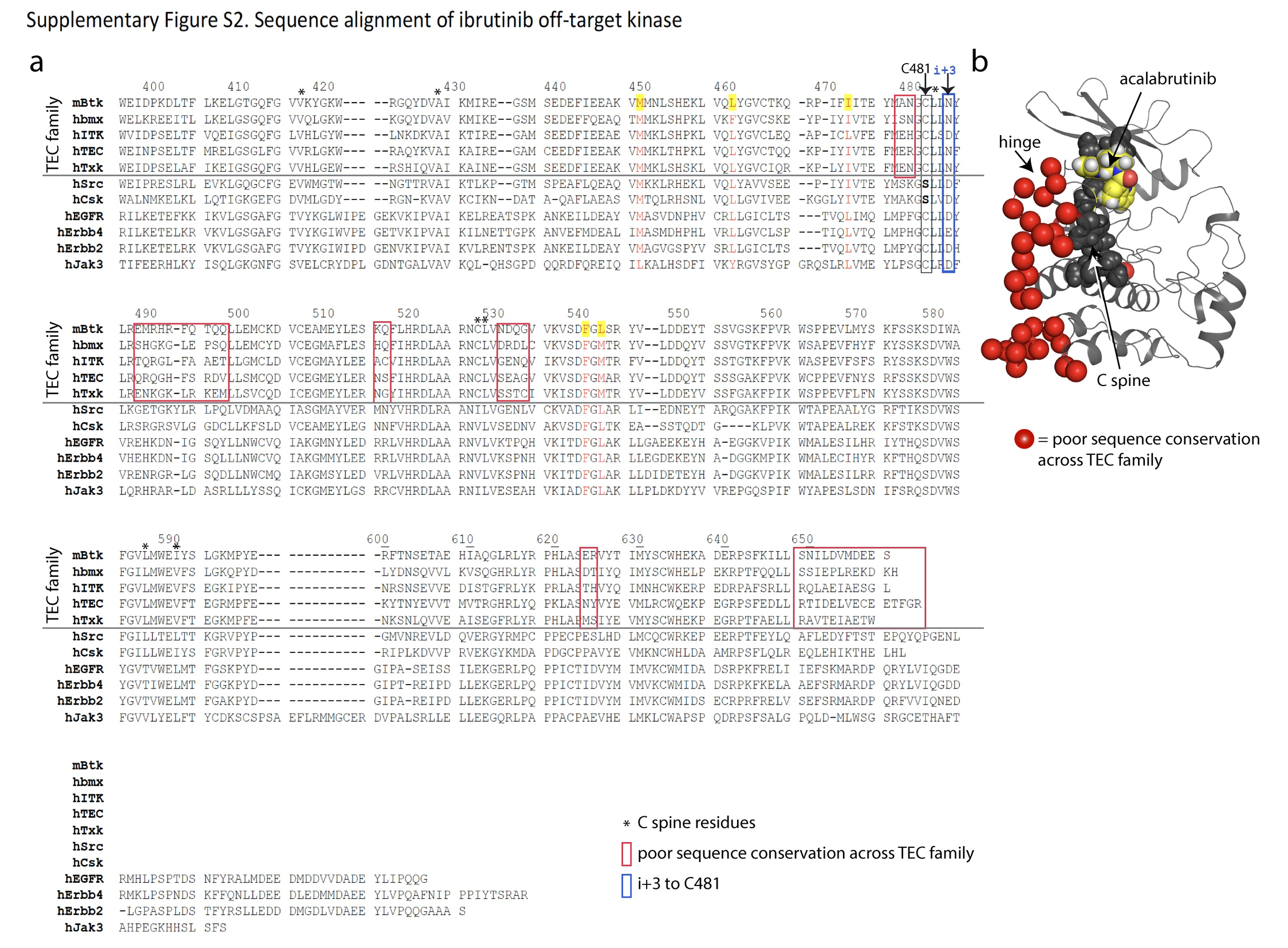

Supplement: S2 Fig — a. Sequence alignment of eleven protein kinases; the five TEC family kinases are listed above the horizontal line. Cys481 (BTK numbering) and the i+3 position are indicated with black and blue boxes, respectively. Residues that surround the active site and directly contact bound drug are in red with yellow highlight. Red boxes indicate regions of low sequence similarity among the TEC kinases. Asterisks indicate C spine residues. b. Structure of the BTK KD/acalabrutinib complex showing the cluster of residues with low sequence conservation in the TEC family (red spheres). Black spheres show the conserved residues of the C spine and bound acalabrutinib is shown in yellow spheres. (TIF) [file pone.0290872.s002.tif]
